# Assessment of Fluorescent Particles for Surface Flow Analysis

**DOI:** 10.3390/s121115827

**Published:** 2012-11-14

**Authors:** Flavia Tauro, Gabriele Mocio, Emiliano Rapiti, Salvatore Grimaldi, Maurizio Porfiri

**Affiliations:** 1 Department of Mechanical and Aerospace Engineering, Polytechnic Institute of New York University, Brooklyn, NY 11201, USA; E-Mails: ftauro01@students.poly.edu (F.T.); rapiti.emiliano@gmail.com (E.R.); 2 Dipartimento di Ingegneria Civile, Edile e Ambientale, Sapienza University of Rome, Rome 00184, Italy; 3 Honors Center of Italian Universities, Sapienza University of Rome, Rome 00184, Italy; 4 Dipartimento per l'Innovazione nei Sistemi Biologici, Agroalimentari e Forestali, University of Tuscia, Viterbo 01100, Italy; E-Mails: gabriele.mocio@libero.it (G.M.); salvatore.grimaldi@unitus.it (S.G.)

**Keywords:** fluorescent particles, hydrologic tracers, sensors technologies, surface flow, velocimetry

## Abstract

In this paper, a systematic performance assessment of the measurement system for surface flow analysis developed by our group in (Tauro et al., Sensors, 2010) is presented. The system is based on the detection of buoyant fluorescent microspheres through a low-cost apparatus, which incorporates light sources to elicit fluorescence response and a digital camera to identify the particles' transit. Experiments are conducted using green fluorescent particles and further tests are executed to evaluate the system performance for red and orange particles varying in emission wavelength, degree of biocompatibility, and cost. The influence of the following parameters on surface flow sensing using fluorescent beads is investigated: (i) distance of the light sources from the water surface, (ii) presence of an ad-hoc filter tuned at the particle emission wavelength, (iii) camera resolution and frame rate, (iv) flow regime, and (v) ambient light. Experimental results are used to inform implementation guidelines for surface flow analysis in natural environments.

## 1. Introduction

Performing accurate and reliable experimental observations is a major challenge in modern hydrology [1,2]. Empirical observations are required to aid the understanding of natural phenomena, validate existing models, and characterize—possibly reduce—uncertainties [3,4]. In this context, considerable efforts should be devoted to the design and development of novel instrumentation as suggested by the International Association of Hydrological Sciences (IAHS) in the “New Scientific Decade 2013–2022” scientific questions [5–16]. For instance, the relevance of surface processes for landscape's evolution [17–20], nutrients' diffusion [21], and human provision [22] has fostered the development of portable optical technologies based on pattern recognition [23–26], reflectivity sensors for buoyant sediments [27], and versatile water tracing systems [28,29]. Nonetheless, current surface flow measurement technologies are affected by a number of factors that pose severe challenges in their practical implementation [30]. Specifically, the accuracy of optical methods can be limited by ambient illumination, light reflections at the water surface, presence of sediments, varying flow regimes, and image distortions due to camera inclination. On the other hand, traditional tracing measurements can be hampered by the need of deploying bulky sensors, significant amount of required tracing material, and presence of operators for physical sampling [31–33].

In this framework, a novel tracing methodology based on the deployment and observation of enhanced fluorescence particles is introduced in [34,35]. This approach seeks to provide accurate estimates of surface flow velocities and travel times by minimizing the amount of tracing material to be released and the complexity of the particle detection equipment. Specifically, such novel tracers are constituted of insoluble and buoyant fluorescent particles of varying diameter, selected based on the specific flow regime. Particles' fluorescence allows for their detection through inexpensive equipment, such as off-the-shelf cameras. Moreover, common illumination sources can be used for eliciting the fluorescent response given the particles' buoyancy. The feasibility of using off-the-shelf green fluorescent particles is thoroughly elucidated in [34–37] by conducting laboratory trials in a miniature water channel, in a natural mountainous stream in the Italian Alps, and in a semi-natural hillslope. Notably, a fast particle tracker is designed in [36] and a low-cost lightweight sensing station, hosting the detection and illumination instruments, is developed in [35,37] for rapid deployment in field studies. These studies demonstrate the applicability of such methodology in the presence of ephemeral micro-channels, fully developed stream flows, water surface reflections, turbid water, and direct sunlight or dim light settings. Yet, a critical analysis of the system performance as the particle emission wavelength is varied along with the testing conditions of the sensing station is lacking.

In this paper, systematic experiments are executed with green-fluorescent particles to assess the performance of the tracing system. In addition, ad-hoc tests are conducted with red-emitting off-the-shelf polyethylene particles and a new class of orange-fluorescent particles, formulated to be environmentally-friendly and biodegradable [38], to explore the effects of alternative fluorophores. Experiments are executed by deploying few grams of particles in a custom-built water channel placed in an outdoor environment and by recording the transit of the beads through the sensing station developed in [35]. Specifically, beads' fluorescence is elicited through commercially available light sources and a miniature digital camera is operated to record the particles' transit. As compared with [35], the sensing station is enhanced to reduce the effect of camera distortions and light reflections at the water surface. Such improvements allow for obtaining higher performance while reducing apparatus' costs. The influence of the following parameters on the system performance is investigated: (i) distance of the light source from the water surface, (ii) presence of a filter at the specific range of emission of the particles, (iii) camera resolution and frame rate, (iv) regime of the investigated flows, and (v) ambient light. Experimental results garnered in this study provide valuable information on the system performance as several key parameters are systematically varied, thus informing implementation guidelines for field studies.

The rest of the paper is organized as follows. In Section 2, the sensing station and the fluorescent particles used for the experiments are presented. In Section 3, descriptions of the experimental setup and protocol are provided. In Sections 4, experimental results are reported and discussed. Conclusions are summarized in Section 5.

## 2. Materials and Methods

### 2.1. Experimental Set Up and Sensing System

**Figure 1.**
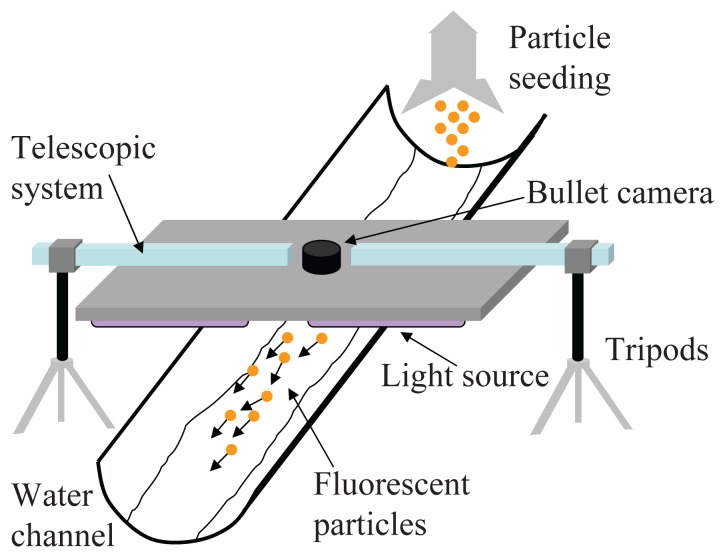
Schematic of the instrumented water channel for surface flow measurements.

The experimental set up is composed of a 2 m long and 22 cm wide reclinable polyvinyl chloride water channel placed outdoor and a portable sensing station, see Figure 1. The water channel cross section is concave and its bed is covered with a mixture of soil and bitumen to create a naturally rough surface. Water is injected into the channel through a hose and a valve is used to regulate flow discharge.

The sensing station is constituted of a 40 cm × 100 cm wooden plate resting on adjustable steel tripods. The tripods allow for raising and lowering the plate at variable distances from the water surface from a few centimeters up to 70 cm. The bottom side of the plate hosts the light unit whose wavelength range is selected to excite the fluorescence of the specific particle tracer. In particular, an array of 14 Ultra Violet (UV) lights in parallel and series connection is used for experiments with green-fluorescent particles and an inexpensive 80 cm strip of LEDs is mounted in series connection below the plate for experiments with both red- and orange-fluorescent beads executed in dim light conditions (direct sunlight is used otherwise). A vertical telescopic system of aluminum bars is connected to the apparatus to hold a calibrating ruler.

**Figure 2.**
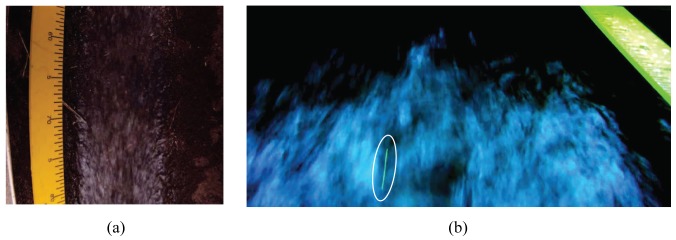
(**a**) Left, image captured with the modified sensing station; (**b**) right, picture taken with the camera offset by the light unit center. The white ellipse in the foreground indicates a deformed green-fluorescent particle.

Differently from [35,37], a miniature water proof Bullet HD 1080p camera is placed in the center of the plate where a circular 2.5 cm aperture is created, see Figure 1. This camera offers performance comparable with much bulkier and more costly devices. Further, such configuration allows for minimizing distortions from the inclination of the camera with respect to the region of interest in the captured videos. For instance, Figure 2(a) depicts the water channel monitored by the sensing station where the camera is placed in the center of the plate. A metric ruler indicates that the image is not severely distorted. On the other hand, Figure 2(b) is recorded with the camera placed offset by the center of the light unit. As emphasized by the metric ruler and the elongated shape of the green-fluorescent particle in the foreground of the picture, the image would require a preliminary orthorectification phase to eliminate distortions and allow for quantitative analysis.

### 2.2. Particle Tracers

In this paper, the performance of the novel measurement system is assessed by conducting experiments with three classes of fluorescent particles.

**Figure 3.**
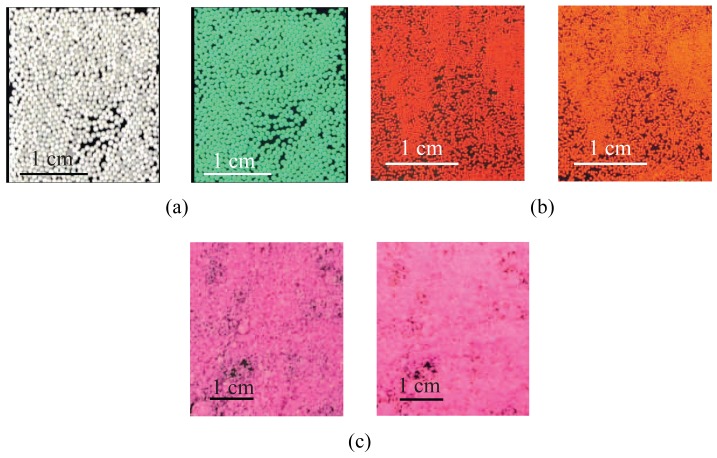
(**a**) Left, view of the green particles under daylight and right, under UV light (365 nm); (**b**) left, view of the red particles under daylight and right, under UV excitation; and (**c**) left, view of the orange particles under daylight and right, under UV light.

Specifically, off-the-shelf 710–1,180 *μ*m yellow-green fluorescent spheres are purchased from Cospheric LLC [39], Figure 3(a). Their cost is 0.8 $/g for 1 kg batches. The beads are white under daylight and emit yellow-green light (561 nm wavelength) if excited by a UV light source (365 nm wavelength). Particles are fabricated by embedding the fluorophore into a polyethylene matrix. Whereas this allows for a lasting and intense luminescence, the deployment of massive quantities of polyethylene can be harmful to the environment. The particles are slightly buoyant, their nominal dry density is 0.98 g/cm^3^, and spherical, thus allowing for enhanced flow tracing performance [36]. A thorough characterization of the visibility of the particles in laboratory controlled conditions, that is, in dark environments and static turbid water, is documented in [34].

Red-fluorescent beads are also purchased from Cospheric LLC, Figure 3(b). Their cost is 9.9 $/g. Such particles are available in smaller sizes, that is, 250–300 *μ*m and their nominal dry density is 0.995 g/cm^3^. They are spherical in shape and present a high emission peak at 605 nm if excited by white light, that is, by a light source emitting from 460 to 650 nm. Such broad excitation range allows for adopting inexpensive and versatile white LEDs as light source in the sensing apparatus. The beads' polyethylene matrix is infused with the fluorophore, thus guaranteeing color-stability.

**Figure 4.**
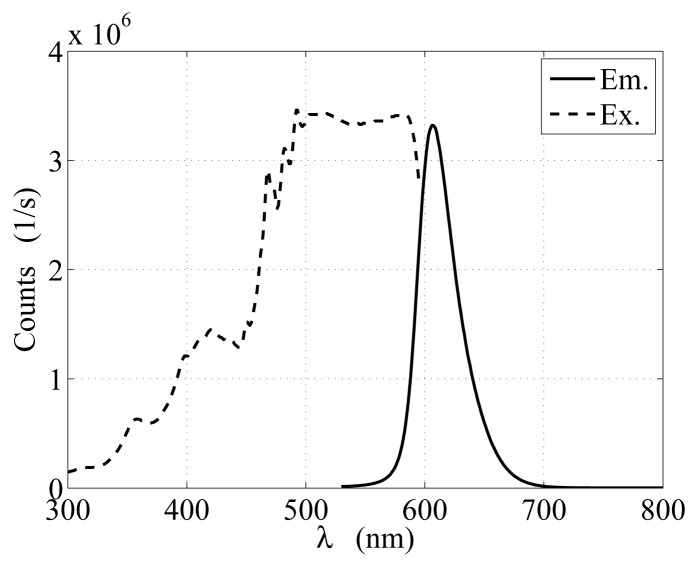
Emission, Em., and excitation, Ex., spectra for the in-house developed orange-fluorescent particles.

Another class of orange-fluorescent particles is considered for experiments with the sensing apparatus, Figure 3(c). Differently from the other tracers, such particles are in-house fabricated from nontoxic Fluorescent FWT Red Dye Concentrate, Cole Parmer^®^, and natural white beeswax pellets purchased from Stakich Inc., MI [38]. The beads are environmentally friendly and their excitation and emission spectra are displayed in Figure 4 as obtained by analyzing a 2 mL sample of melted particles with PTI Quanta Master 40 spectrofluorometer. The particles are produced by melting the beeswax at 60°–65° C and then mixing it with a 6 × 10^−3^*μ*g/L diluted solution of the fluorophore. The homogeneous emulsion of beeswax and fluorophore is then instantaneously cooled down by adding water at 5 ° C. This phase leads to the formation of wax drops of variable diameters that rapidly solidify and migrate to the water surface of the suspension. Beads of 250–420 *μ*m in diameter are obtained through filtering and sieving the material. The particles are neutrally buoyant and spherical in shape and their cost is only 0.025 $/g.

### 2.3. Data Processing

Image analysis tools developed in [34] are used to identify the transit of the particles in the water channel. Specifically, videos of the particles are converted to RGB frames and the green and red channels for the green and red- and orange-fluorescent particles, respectively, are analyzed by using the index 𝒢 defined by [35], that is,

(1) $$\begin{array}{cc} 𝒢=\frac{\sum_{i\in 𝒥}c_{i}^{\alpha }n_{i}}{\sum_{i\in 𝒥}n_{i}}, & 𝒥=\{i\in \{0,1,\ldots ,255\}:n_{i}>0\}, \end{array}$$

with $n_{i}=n_{i}^{p}-n_{i}^{b}$. Here, $n_{i}^{b}$ and $n_{i}^{p}$ refer to the pixel count for the background and particle images, respectively. The term $c_{i}^{\alpha }$ represents the intensity classes from 0 to 255 where the power *α* is introduced to emphasize brighter pixels that are likely to correspond to the fluorescent beads. Specifically, the value of the power is selected by performing preliminary analyses where *α* is varied from a low value of 2 to 15. It is found that high values are necessary to emphasize the presence of a meagre number of highly fluorescent particles, whereas lower *α* values are appropriate in case of numerous particles emitting at lower intensities. Background images are obtained from the original ones by either applying a bottom-hat transformation [40,41] or by starting videos before particle deployment and then selecting initial frames. The introduced index 𝒢 is computed on the cropped images where the only water surface is captured. This procedure allows for identifying the brightest frame sequences in recorded videos, thus detecting the beads' transits.

## 3. Experimental Procedure

Experiments are executed by deploying batches of approximately 3 g of fluorescent particles in the water channel. Surface flow velocity in the channel is first experimentally measured by deploying corks and other similar lightweight objects on the water surface and then evaluating the time they take to flow through the channel with a chronometer. The sensing apparatus is placed across the channel with the light unit and camera parallel to the water surface, see Figure 1.

Experiments are conducted to investigate the variation of the system performance to the following factors: distance of light unit and camera from the water surface, resolution and frame acquisition rate of the camera, flow velocity, outdoor illumination conditions, and presence of an optical filter to isolate particle emission wavelength. Specifically, tests are performed with the green-fluorescent particles by adjusting the distance of the plate from the water surface, *H*, to 15, 40, or 70 cm in a decreasing level of illumination. For each distance, the resolution of the Bullet camera is set to Full HD, that is, 1,920 × 1,080 pixels with acquisition frequency to 30 fps or to WVGA, that is, 848 × 480 pixels with acquisition frequency to 60 fps. Further, the surface flow velocity is set to 0.5, 1, or 2 m/s in an increasing level of severity of the particle detection. Each set of experiments is performed both in the morning and in the afternoon, (labeled “Morning” and “Afternoon”, respectively, in Section 4). Moreover, experiments are performed with or without a 568 nm optical filter before the camera lens. Its cost is approximately $56. Two additional experiments are executed at night, which is expected to be the best illumination condition for the maximal contrast of fluorescent particles against the background (labeled “Night” in Section 4). These conditions address the most challenging experiments at the higher distances of the sensing station from the water surface and the fastest velocity. Full HD resolution is used in these tests and filters are not employed.

A total of 74 experiments are performed with the green-fluorescent particles to provide a thorough characterization of the methodology. Experimental conditions in which green particles are not detected are executed with the red- and orange-fluorescent particles to compare tracers' performance and provide guidelines for experiments in adverse outdoor conditions and natural settings. Specifically, tests are conducted at distances of the light unit equal to 40 and 70 cm above the water surface, for the highest velocity of 2 m/s, without filter, Full HD and WVGA resolutions, and direct sunlight conditions. Also, two experiments are performed at night setting *H* = 70 cm, *v* = 2 m/s, and Full HD resolution and without filter.

## 4. Results and Discussion

### 4.1. Green-Fluorescent Particles

**Figure 5.**
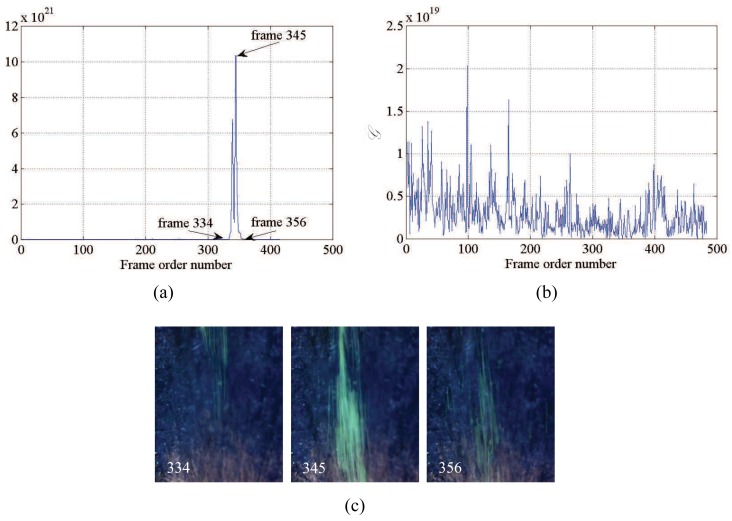
(**a**) Time series of the index 𝒢 for a successful experiment; (**b**) time series of the index 𝒢 for a failed experiment; and (**c**) frames depicting the entrance, transit, and exit, respectively, of the particles in the region of interest for the successful experiment in (a).

Findings from experiments conducted with the green-fluorescent beads are reported in Table 1, where letter “Y” indicates successful experiments and letter “N” denotes failed tests. Specifically, experiments are considered successful if the time series of the index 𝒢 presents a clearly identifiable peak and such peak depicts the transit of the particles in the field of view from visual inspection. For reference, Figure 5(a) shows a successful experiment conducted in the morning, at *H* = 15 cm, *v* = 2 m/s, WVGA resolution, and without filter; whereas Figure 5(b) displays a failed experiment under more severe conditions, that is, recorded during the morning, at *H* = 40 cm, *v* = 2 m/s, WVGA resolution, and without filter. Specifically, intensity values in Figure 5(b) are generally lower than intensities in Figure 5(a) and observed peaks do not correspond to the transit of the particles in the region of interest. As reported in Figure 5(c), frames 334, 345, and 356 correspond to the entrance, the transit, and the exit of the cloud of fluorescent particles underneath the camera, respectively. As expected, frame 345 depicts the maximum presence of beads in the captured field of view.

Labels in Table 1 report the settings at which each experiment is conducted. Specifically, tests are conducted for three values of *H* and *v*, in Morning and Afternoon illumination conditions, by varying camera resolution from Full HD to WVGA, and by using a 568 nm optical filter or the sole Bullet camera. All experimental tests are successful for *H* = 15 cm under both Morning and Afternoon illumination conditions and at *H* = 40 cm and *H* = 70 cm for Afternoon light settings. On the other hand, tests performed in Morning settings at *H* = 40 cm and *H* = 70 cm for *v* = 1 m/s and *v* = 2 m/s do not lead to the detection of the particles. The experiment in Night settings for *H* = 40 cm is successful while increasing *H* further hampers particles' detection.

The results of this study indicate that outdoor illumination is a crucial parameter for the visibility of green-fluorescent particles. Specifically, bright illumination of Morning settings tend to increase reflections at the water surface, thus resulting in noisier time series for 𝒢. The effect of intense sunlight also abates the low power UV light excitation, and therefore the beads appear whiter and harder to distinguish against the background. This finding is evident for the higher water speeds, where experiments executed in Morning settings do not yield successful results. On the other hand, the effect of the filter is marginal for particle detection. As documented in Table 1, the presence of the filter does not improve the visibility of the beads under varying light conditions. Therefore, the use of a filter can likely be avoided to enhance the portability of the station and reduce its cost.

**Figure 6.**
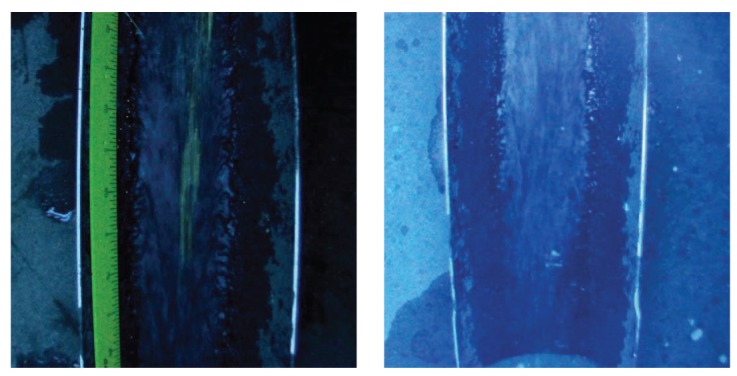
Left, picture of the visibility test conducted in the afternoon at *H* = 70 cm, *v* = 2 m/s, Full HD resolution, and without filter. Right, picture of the visibility test conducted at night at *H* = 70 cm, *v* = 2 m/s, Full HD resolution, and without filter.

Failure of the Night experiment conducted by setting *H* = 70 cm, *v* = 2 m/s, Full HD resolution, and without filter is likely due to the diffused UV light that creates a uniform nuance in the pictures and decreases the contrast of foreground objects against the background. Figure 6 compares pictures acquired for similar settings, that is, *H* = 70 cm, *v* = 2 m/s, Full HD resolution, and without filter, but different illumination conditions. It is observed that the frame captured in Afternoon conditions, Figure 6 left, presents higher contrast with respect to the frame acquired at night, Figure 6 right, where the UV lights tend to create a uniform blue-violet background.

### 4.2. Red- and Orange-Fluorescent Particles

Results for experiments conducted on the green-fluorescent particles highlight the following drawbacks: the particles are not clearly detectable in Morning light conditions for *H* equal or greater than 40 cm and *v* equal or greater than 1 m/s with both Full HD and WVGA camera resolutions and the beads are not visible at *H* = 70 cm and *v* = 2 m/s for Night light conditions due to low contrast in the pictures. Experiments under such adverse conditions are replicated with red- and orange-fluorescent particles to test the performance of alternative fluorophores. Specifically, experiments are executed for the fastest flow velocity of 2 m/s and for the higher distances of the camera and light units from the water surface, that is, 40 and 70 cm, see Table 2. Tests conducted in Morning conditions are performed under direct sunlight to increase the complexity of the tests. Additional experiments are conducted at night consistently with the failed test in Table 1 with the green particles. Optical filters are not used throughout the entire set of experiments with the red- and orange-fluorescent particles as their use is found to be marginally relevant in experiments on green beads.

The red-fluorescent beads demonstrate improved performance in severely illuminated conditions as compared with the green particles, see Table 2. In particular, experimental results indicate that particles can be detected for *v* = 2 m/s and *H* = 40 and 70 cm even under direct sunlight (Morning illumination conditions without external lamps) provided that a Full HD camera is used. The need for using Full HD camera is likely due to the small size of the particles, 250–300 *μ*m, which limits the implementation of a WVGA camera. The visibility of such small particles is also particularly challenging due to the effect of high reflections from the direct sunlight illumination conditions.

Such promising finding suggests that the use of red-fluorescent beads may lead to simpler and less expensive sensing stations for particle detection in complex and remote environments. Possibly, inexpensive strip-LEDs could be used as fluorescence excitation source in the absence of external illumination as demonstrated by the successful test achieved at night and *H* = 70 cm.

As displayed in Table 2, good results are also obtained with the orange-fluorescent particles. In particular, the beads can be detected in Morning conditions, without external lamps, *v* = 2 m/s, *H* = 40 cm, and Full HD camera resolution and even at *H* = 70 cm at night. Consistently with experiments on red particles, Full HD resolution tends to be beneficial given the minimal size of the beads, that is, 250–420 *μ*m. On the other hand, the fluorescence of the particles is not appreciable under direct sunlight for the highest distance and with Full HD camera resolution. This can be attributed to the fact that the intensity of the emission is weaker than the fluorescence obtained with the red beads.

### 4.3. Remarks

A comprehensive summary of findings garnered from the experimental analysis is presented in Table 3 for Morning and Night illumination conditions.

Under direct sunlight, the red-fluorescent particles offer high performance at relevant distances of the light and video units from the water surface, whereas the green beads are not visible beyond 20 cm from the camera. Notably, the former particles do not require any external lamp to elicit fluorescence. Further, the presence of the filter is not beneficial under Morning and Night conditions and, therefore, filters can be dispensed with in the sensing station. On the other hand, camera resolution is required to be high for both settings and for each class of particles. While the need of high resolution can be attributed to the minimal size of the red and orange particles, such setting demonstrates the lower performance of the green-fluorescent beads. In addition, green particles cannot be detected in case of high flow velocity and severe light conditions. Each class of particles presents improved visibility in Night conditions, when the effect of reflections at the water surface is reduced. Therefore, findings in Table 3 demonstrate that the red beads tend to be the most versatile and effective for surface flow tracing. Nonetheless, orange-fluorescent beads can be deemed as valid alternatives for flow monitoring due to their promising performance, low cost, and environmental compatibility.

## 5. Conclusions

In this paper, systematic experiments are conducted with green-fluorescent particles to determine the performance of the sensing system developed in [35,37] for surface flow measurements in natural environments. Tests are also executed to explore the effects of alternative particle emission wavelengths on the tracing system efficiency. Further, the sensing station used in previous prototypes is technically improved to enhance the portability, reduce the costs, and mitigate detrimental effects due to external illumination conditions.

Experimental findings demonstrate that red-fluorescent off-the-shelf particles have superior performance under multiple conditions. Differently from green-fluorescent particles, red beads are clearly visible under direct sunlight at relevant distances from the camera and fast flow velocities. Similar results are obtained using environmentally friendly particles that emit in the orange spectrum. In particular, red and orange fluorophores demonstrate enhanced performance due to their broad excitation spectra, whereas they are minimally affected by environmental conditions such as low water temperature and presence of sediments. Successful experiments are conducted for distances of the camera and light units from the water surface of up to 40 cm and flows of 2 m/s. It is expected that adjusting the camera acquisition frame rate would allow for experimenting with more severe flow rates, such as mountainous stream velocities. On the other hand, the intensity of the bead emissions is not sufficient for detecting the particles at higher distances under direct sunlight.

Future research will aim at exploring alternative nontoxic fluorophores for the environmentally friendly particles to increase emission intensity under white light excitation. This will allow for adopting inexpensive LEDs as fluorescence excitation source or possibly using sunlight radiation. The miniaturization of the light source will leverage the design and fabrication of affordable and lightweight sensing stations for implementation in engineering practice.

## Figures and Tables

**Table 1.** Summary of the green-fluorescent particle experiments.

		*H* = 15 cm				*H* = 40 cm					*H* = 70 cm				
		Morning		Afternoon		Morning		Afternoon		Night	Morning		Afternoon		Night
		Full HD	WVGA	Full HD	WVGA	Full HD	WVGA	Full HD	WVGA	Full HD	Full HD	WVGA	Full HD	WVGA	Full HD
*v* = 0.5m/s	Filter	Y	Y	Y	Y	Y	Y	Y	Y		Y	Y	Y	Y	
	No Filter	Y	Y	Y	Y	Y	Y	Y	Y		Y	Y	Y	Y	
*v* = 1m/s	Filter	Y	Y	Y	Y	N	N	Y	Y		N	N	Y	Y	
	No Filter	Y	Y	Y	Y	N	N	Y	Y		N	N	Y	Y	
*v* = 2m/s	Filter	Y	Y	Y	Y	N	N	Y	Y		N	N	Y	Y	
	No Filter	Y	Y	Y	Y	N	N	Y	Y	Y	N	N	Y	Y	N

**Table 2.** Summary of the red- and orange-fluorescent particle experiments.

			*H* = 40cm		*H* = 70cm		
			Morning		Morning		Night
			Full HD	WVGA	Full HD	WVGA	Full HD
Red	*v* = 2m/s	No Filter	Y	N	Y	N	Y
Orange	*v* = 2m/s	No Filter	Y	N	N	N	Y

**Table 3.** Summary of the optimal testing conditions for the sensing station under varying light settings.

	Morning	Night
Green beads	*H* = 15 cm	*H* marginal
	No filter	No filter
	Full HD	Full HD
	Slow flows	*v* marginal

Red beads	*H* marginal	*H* marginal
	No filter	No filter
	Full HD	Full HD
	*v* marginal	*v* marginal

Orange beads	*H* = 40 cm	*H* marginal
	No filter	No filter
	Full HD	Full HD
	*v* marginal	*v* marginal
